# Surgical Notes

**Published:** 1860-07

**Authors:** E. Andrews

**Affiliations:** Professor of Surgery in the Medical Department of Lind University


### SURGICAL NOTES.

By E. ANDREWS. M. D.,
Professor of Surgery in the Medical Department of Lind University

*Dislocation of the Hip. Reduction by Reid's Method.*—Anthony B. was admitted to Mercy Hospital on the night of the 8th of June. While driving a load of lumber, the wagon upset, and he was picked up with a contusion of the left knee and a dislocation of the corresponding hip. He was laid in an ox wagon and brought to the city, a distance of twelve miles, suffering excruciating torture from the motion of the vehicle. On examination, I found the limb rotated inward, the great toe pointing to the hollow of the opposite foot, and firmly resisting any attempt to rotate it outward. There was very little shortening, and the trochanter was removed somewhat too far from the spinous process of the ilium. In short, it proved to be a dislocation into the Sciatic Notch.

My theory of the way in which the accident occurred, was, that in falling he struck upon the knee while the thigh was flexed and abducted, and that the same blow which caused the contusion drove the head of the bone out through the posterior part of the capsular ligament.

After administering a mixture of chloroform and ether to full anæsthesia, I reduced the dislocation by manipulation, or as it is termed sometimes, by "Reid's method," and it returned readily, and with the usual "jerk," to its socket. The patient recovered rapidly.

---

*Neuralgia of the Face of thirteen years standing. Cure by removing a portion of the nerve.*—J.——B.——, of Kankakee, had suffered, by his account, thirteen years, with a most terrible neuralgia in the lower part of the right side of his face. The pain seemed to recur in paroxysms every three or four minutes, when he would be incapable of attending to anything but his extreme torture, and endeavor to alleviate a little his agony by rubbing the part briskly with his hand until the paroxysm subsided.

On examination of the case, I discovered that the pain was limited exclusively to the nerves which make their exit from the right anterior mental foramen. I judged, therefore, that the cause of the trouble was at that opening, and that a section of the nerve on the proximal side of it would relieve the pain. I was the more willing to adopt this course because the patient had already exhausted almost every other resource in vain, and his suffering was so terrible that the paralysis of the nerve, by removing a portion, was comparatively a trivial evil. I therefore administered chloroform and ether to anæsthesia, and operated as follows: I made a semi-circular incision over the masseter muscle down to the ramus of the jaw, with the convexity of the incision downward. Dissecting the flap upward, the external surface of the ramus of the jaw was laid bare, and the trephine applied. If a line be drawn from the angle of the jaw upward and forward to a point half an inch behind the last molar tooth, and the centre pin of a half inch trephine be placed upon this line at such a point that the crown of the instrument is a quarter of an inch from the anterior edge of the ramus, the nerve will be found crossing the centre of the opening made by the instrument. I have found this direction rather more convenient than those laid down by our authors. When the crown of the trephine cuts with the whole circumference in cancellar tissue, it may be withdrawn, and the button of bone removed by the broad end of an elevator. This leaves the inner table of bone intact, and does not seriously weaken an adult jaw. Half an inch of the nerve having been removed with the scissors, and the hæmorrhage stopped by pressure, the wound was closed up. For twenty-four hours the patient suffered, instead of the paroxysmal torture which previously affected him, a steady pain which he referred to the parts supplied by the severed trunk; after that, to his great delight, he found himself free from all suffering. The wound healed well and the patient was discharged.

---

*Lithotomy. Opthalmia in Southern Illinois,*—S.—–M.—–, residing in the southern part of Illinois, aged 11 years, had for a period of many months all the usual symptoms of a urinary calculus. His physician, Dr. Mitchell, of Attilla, having examined the case, discovered a calculus of considerable size, and requested me to remove it. For this purpose I placed the patient under the influence of ether and chloroform, equal parts, and performed the usual lateral operation. The calculus was cylindrical, and about one inch in thickness by two and a half inches in length. The two ends were curiously curved, and the surface very rough. The case convalesced remarkably well under the care of Drs. Mitchell and Jewell, and the recovery was perfected without accident.

While making the journey for this operation, as also on several other occasions, I had an opportunity to observe the extreme frequency of opthalmia in the southern counties of Illinois. The disease in its acute stage seems to be very often of an erysipeloid character, and rapidly assuming the purulent form, destroys vision in great numbers of cases. In other instances, where vision is not destroyed, there often remains a tedious chronic inflammation, followed by opacity of the cornea, cataract, entropium, or other forms of disease.

I have not been able to learn any local cause for the prevalence of this trouble. Will not some of our southern bretheren give us the results of their investigations on the subject?

